# Dynamic composition, shaping and organization of plastid nucleoids

**DOI:** 10.3389/fpls.2014.00424

**Published:** 2014-09-04

**Authors:** Marta Powikrowska, Svenja Oetke, Poul E. Jensen, Karin Krupinska

**Affiliations:** ^1^Department of Plant and Environmental Sciences, VILLUM Research Centre for Plant Plasticity and Copenhagen Plant Science Centre, University of CopenhagenCopenhagen, Denmark; ^2^Plant Cell Biology, Institute of Botany, Christian-Albrechts-University of KielKiel, Germany

**Keywords:** chromatin, nucleoid, plastid DNA, ptNAP, thylakoids

## Abstract

In this article recent progress on the elucidation of the dynamic composition and structure of plastid nucleoids is reviewed from a structural perspective. Plastid nucleoids are compact structures of multiple copies of different forms of ptDNA, RNA, enzymes for replication and gene expression as well as DNA binding proteins. Although early electron microscopy suggested that plastid DNA is almost free of proteins, it is now well established that the DNA in nucleoids similarly as in the nuclear chromatin is associated with basic proteins playing key roles in organization of the DNA architecture and in regulation of DNA associated enzymatic activities involved in transcription, replication, and recombination. This group of DNA binding proteins has been named plastid nucleoid associated proteins (ptNAPs). Plastid nucleoids are unique with respect to their variable number, genome copy content and dynamic distribution within different types of plastids. The mechanisms underlying the shaping and reorganization of plastid nucleoids during chloroplast development and in response to environmental conditions involve posttranslational modifications of ptNAPs, similarly to those changes known for histones in the eukaryotic chromatin, as well as changes in the repertoire of ptNAPs, as known for nucleoids of bacteria. Attachment of plastid nucleoids to membranes is proposed to be important not only for regulation of DNA availability for replication and transcription, but also for the coordination of photosynthesis and plastid gene expression.

## Introduction

Plastids are the characteristic organelles of photosynthetic eukaryotes. They are the sites of photosynthesis, and their biosynthetic pathways supply the plant cell with many essential compounds. Chloroplasts evolved from a cyanobacterial ancestor after a single endosymbiotic event, that was followed by an extensive reduction of the plastid genome size (Timmis et al., 2004; Bock and Timmis, 2008; Green, 2011). Among the genes still present in the 100–200 kbp plastid genomes are the ribosomal RNA genes, 27–31 genes encoding tRNAs, and a variable number of other genes, that in higher plants include about 85 encoding proteins of the photosynthetic apparatus (Green, 2011).

Within the chloroplast, multiple copies of the plastid DNA (ptDNA) together with RNA and proteins are organized in structures that are similar to bacterial nucleoids. The compact structure of DNA in such nucleoids has been compared with the chromatin in the nucleus of eukaryotic cells (Sakai et al., 2004). The fundamental difference between genome organization in plastids vs. that in bacteria is, that plastids have multiple nucleoids with a varying number of genome copies, whereas bacteria only have a single nucleoid containing a variable number of DNA molecules. Nucleoids contain all enzymes necessary for transcription, replication and segregation of the plastid genome (Sakai et al., 2004). Moreover, posttranscriptional processes including RNA splicing and editing, as well as ribosome assembly, take place in association with the nucleoid, suggesting that these processes occur co-transcriptionally (Majeran et al., 2012). However, among the many proteins found in the nucleoid and identified by proteomic analyses (Phinney and Thelen, 2005; Majeran et al., 2012; Melonek et al., 2012) only a few have been functionally characterized so far. In Table 1, proteins, that were proposed to play roles in nucleoid architecture, and which in analogy to the architectural proteins of bacterial nucleoids have been named plastid nucleoid associated proteins (ptNAPs) (Krupinska et al., 2013), are listed.

**Table 1:** **Characteristics of plastid nucleoid associated proteins proposed to be involved in shaping and organization of nucleoids in plants**.

**Name**	**MW theoretical**	**pI**	**Proposed function**	**Occurrence/Species characterized**	**References**
PEND, plastid envelope DNA binding protein	130 kDa	4.6^#^ (10.3^*^)^#^	anchoring of nucleoids to the envelope membrane	dicots/*Pisum sativum*, *Brassica napus*	Sato et al., 1993, 1998; Sato and Ohta, 2001; Terasawa and Sato, 2005; Wycliffe et al., 2005
MFP1, MAR-binding filament-like protein	90 kDa	8.5	anchoring of nucleoids to thylakoids	angiosperms/*Lycopersicum temulentum*	Meier et al., 1996; Jeong et al., 2003, 2004
TCP34, (tetratricopeptide-containing chloroplast protein)	38 kDa	5.4^#^	candidate nucleoid anchoring protein	higher plants/*Spinacia oleracea*	Weber et al., 2006
SWIB-4, domain of SWI/SNF complex B	12 kDa	10	packaging of DNA	angiosperms/*Spinacia oleracea, Arabidopsis thaliana*	Melonek et al., 2012
pTAC3^**^	68 kDa^#^	4.6^#^ (9.6)^#^	candidate DNA packaging protein	land plants except gymnosperms/*Zea mays*	Majeran et al., 2012
SiR (DCP68), sulfite reductase (DNA compacting protein)	68 kDa	9.1^#^	bifunctional: DNA compaction and sulfur assimilation	cyanobacteria, algae and land plants/*Glycine max, Pisum sativum, Zea mays*	Cannon et al., 1999; Sekine et al., 2002, 2007, 2009; Sato et al., 2003; Kang et al., 2010; Wiedemann et al., 2010
YlmG	23 kDa	10.9	nucleoid partitioning	cyanobacteria and plastid containing eucaryotes, *Arabidopsis thaliana*	Kabeya et al., 2010
SVR4/-like (MRL7/-like), suppressor of variegation 4	28 kDa	5.2^#^	putative chaperones for NAPs	mosses, clubferns and angiosperms, *Arabidopsis thaliana, Hordeum vulgare*	Qiao et al., 2011; Yu et al., 2011; Yua et al., 2013; Powikrowska et al., 2014
pTAC16^**^	54 kDa	8.9^#^	putative membrane-anchor	angiosperms/*Arabidopsis thaliana*	Ingelsson and Vener, 2012
WHIRLY1, 3 (pTAC1, pTAC11)^**^	24-26 kDa	9.3^#^	condensation of DNA of a subgroup of nucleoids	angiosperms/*Arabidopsis thaliana, Zea mays, Hordeum vulgare*	Pfalz et al., 2006; Prikryl et al., 2008; Maréchal and Brisson, 2010; Krupinska et al., 2014

*MW, molecular weight; pI, isoelectric point*.

* *pI of the proteins basic region*.

# *pI or protein molecular weight was determined with ExPASy Protparam (http://web.expasy.org/cgi-bin/protparam/protparam)*.

** *pTAC, protein detected in the transcriptional active chromosomes of chloroplasts from Arabidopsis thaliana (Pfalz et al., 2006)*.

The dynamic shaping of nuclear chromatin and bacterial nucleoids is known to have profound effects on gene expression. Whereas the mechanisms underlying chromatin remodeling in the nucleus of plant cells have been investigated intensively, research on the mechanisms underlying the dynamics of the structure and organization of plastid nucleoids is still in its infancy. This is in sharp contrast with the enormous importance of chloroplast metabolism for growth and productivity of plants. Expression of plastid genes needs to be continuously coordinated with the activity of the nuclear genome. Structural changes are likely to be involved in the crosstalk between plastid and nuclear genomes.

In this article recent progress in the elucidation of the composition of plastid nucleoids is reviewed in the context of the complex DNA-protein architecture. The unique characteristics of plastid nucleoids will be highlighted by comparison with bacterial nucleoids and nuclear chromosomes. The involvement of plastid specific NAPs in regulation of DNA availability for replication and transcription and the functional significance of nucleoid association with the thylakoid membranes in chloroplasts will be discussed.

## Microscopic analyses of plastid nucleoid morphology

**Figure 1:**
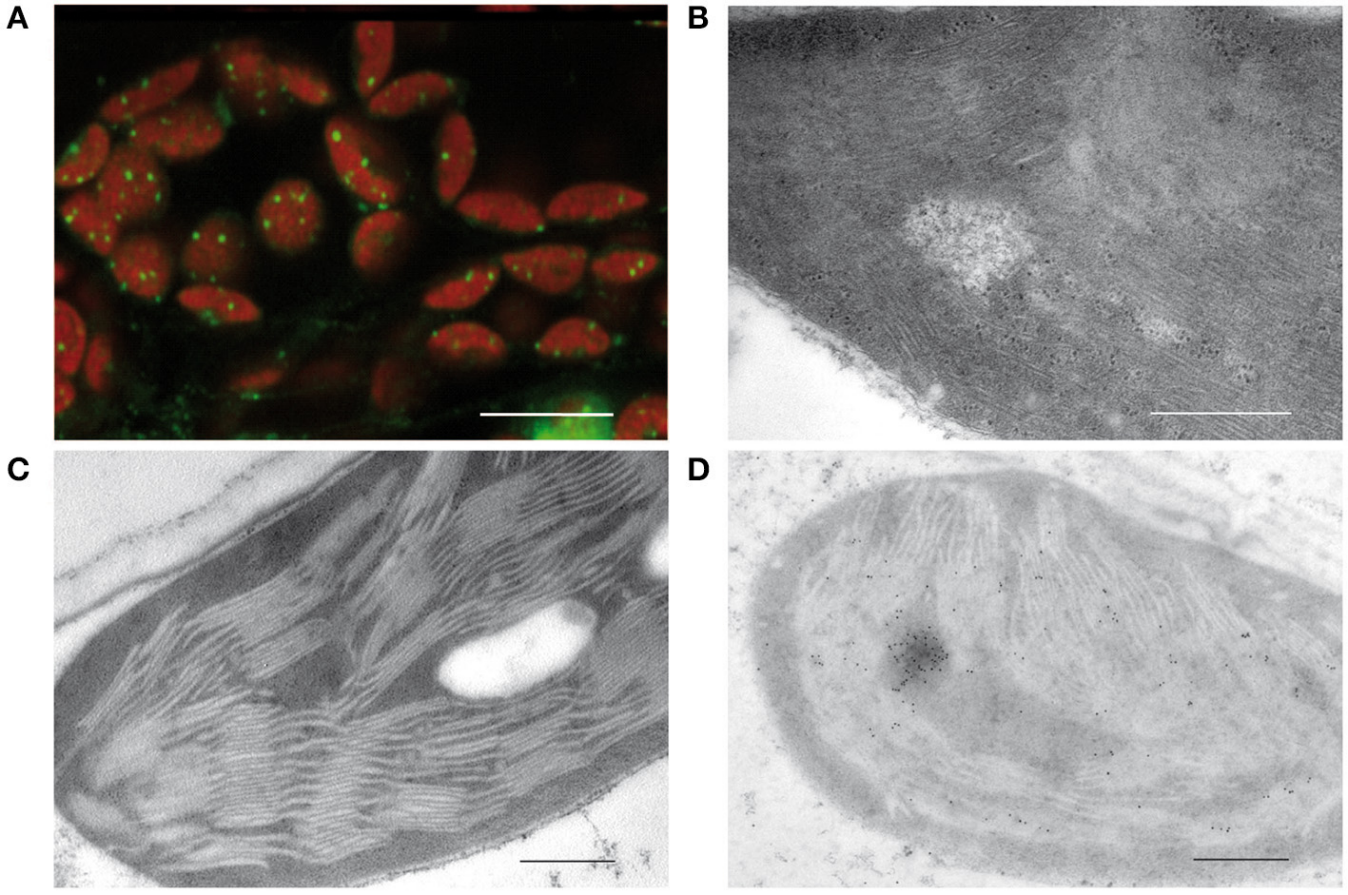
**Visualization of plastid nucleoids by using different microscopic techniques. (A)** Nucleoids visualized by fluorescence microscopy of SYBR Green in leaf sections, bar: 10 μm. **(B)** Conventional electron micrographs showing nucleoids with DNA filaments in mesophyll chloroplasts. **(C)** Specimen prepared by high pressure freezing and freeze substitution (HPF-FS). **(D)** Immunogold labeling of nucleoids in leaf sections obtained from specimen prepared by high pressure freezing and freeze substitution (HPF-FS) using a DNA specific antibody, bar: 500 nm.

In 1962, Ris and Plaut discovered irregularly shaped bodies containing DNA in the chloroplast of Chlamydomonas by staining with acridine orange. Electron micrographs revealed microfibrils in areas of low density corresponding to DNA macromolecules similar to those that were shown before in bacteria (Robinow and Kellenberger, 1994). These microfibrils suggested that at least part of the plastid DNA is “naked” in contrast to the nuclear DNA that together with basic proteins, histones, is organized in highly compact structures known as chromatin (Kuroiwa, 1991). Images obtained by staining with 4′,6-diamidino-2-phenylindole (DAPI) or other DNA dyes such as SYBR Green revealed a quite different organization of plastid DNA. In chloroplasts, tiny compact structures associated with the thylakoids are detectable (Figure 1A). Protease treatment and reconstitution assays on such isolated structures indicated that the packaging degree of DNA is higher than in the metaphase chromosomes of animals (Nemoto et al., 1988; Kuroiwa, 1991). From these results it was concluded that ptDNA is not “naked,” but tightly packed in nucleoids by interactions with basic proteins as it is also known for the nuclear chromatin.

Indeed, the concept of “naked” DNA in plastids and bacteria was based only on conventional electron microscopy employing chemical fixation and dehydration of the tissue, known to lead to denaturing and loss of proteins. As a result, DNA filaments devoid of proteins get visualized in electron-lucent areas from which proteinous material was lost during dehydration (Figure 1B). When instead of chemical fixation, physical fixation by high pressure freezing and freeze substitution (HPF-FS) is employed, no DNA filaments are detectable (Figure 1C). Specimens prepared by HPF-FS were used for immunogold labeling with an antibody specific for single- and double-stranded DNA. Thereby regions of intensive labeling could be detected that have about the size of nucleoids as detected by epifluorescence or confocal microscopy (Figure 1D).

## DNA organization and gene expression in the nucleus

Genomic DNA in most eukaryotic cells is hierarchically organized within the chromatin (Campos and Reinberg, 2009; Fudenberg and Mirny, 2012). The basic unit of chromatin is the nucleosome that consists of double stranded DNA wrapped around a histone octamer. The nucleosomes organize into 11 nm fibers that resemble beads on strings. This structure is thought to further fold into so-called 30 nm fibers stabilized by the H1 linker histone. Although very little is known about the organization of chromatin beyond this stage, it is assumed that organization of the higher order chromatin structure involves formation of interacting fibers, chromatin loops and positioning to generate a distinctive spatial arrangement of the genome within the three-dimensional space of the nucleus (for a review see Li and Reinberg, 2011).

In general, the higher-order structures of nuclear chromatin inhibit DNA transaction processes, i.e., replication, repair, recombination and transcription of the DNA (Li and Reinberg, 2011). These DNA transaction processes require chromatin remodeling by mechanisms such as: (i) posttranslational modifications (acetylation and methylation) of N- and C-terminal tails of histones, (ii) exchanging histones variants, (iii) DNA methylation, (iv) non-histone architectural proteins, (v) ATP-dependent nucleosome remodelers, as well as (vi) the action of negatively charged histone chaperones.

Most eukaryotic genes are transcribed by RNA polymerase II (RNAP II). Interestingly, transcription by RNA polymerase II requires dynamic changes in the chromatin structures of the templates (Orphanides and Reinberg, 2000; Studitsky, 2005). During high rates of transcription, nucleosomes are completely disassembled and reassembled with the assistance of ATP-dependent nucleosome remodelers and histone chaperones altering contacts between DNA and histones. These remodelers are specific for certain genes in different cell types and contexts of cell differentiation (de la Serna et al., 2006). ATP-dependent nucleosome remodelers allow the DNA to “inch-worm” around the histone octamer. Acidic histone chaperones, on the other hand, “collect” the basic histones after the histone-DNA interactions have been broken by the ATP-dependent nucleosome remodelers.

Non-histone architectural proteins, such as high mobility group (HMG) proteins (Grasser, 1995) also play a role in chromatin structural dynamics, since they decrease the compactness of the chromatin fiber and enhance the accessibility of DNA to regulatory factors. Members of the HMGN family contain a functional nucleosome-binding domain (NBD) and a negatively charged C-terminus of varying length. It has been shown that the negatively charged C-terminal domain of HMGN5 interacts with the positively charged C-terminal domain of the linker histone H1 and thereby counteracts the H1-mediated compaction of a nucleosomal array. In turn, this facilitates transcriptional activation (Rochman et al., 2010).

Packaging of DNA by histones into nucleosomes is not a distinguishing feature of eukaryotes, but also occurs in some groups of archaebacteria which might have participated in the origin of eukaryotes (Bendich and Drlica, 2000). In any case, a nucleosome based packaging of DNA results in a rather closed structure, and the access of DNA by DNA transaction enzymes involves several interconnected processes modeling the chromatin.

## DNA organization and gene expression in bacteria

Whereas the ability of histones to interfere with the nuclear chromatin structure and thereby to regulate transcription is rather well conserved among eukaryotes and understood in great detail, the situation in eubacteria seems to be more diverse and complicated. Research on the folding of bacterial DNA began in the 1970s, but the first systematic inventory of nucleoid associated proteins (NAP) (Azam and Ishihama, 1999) is still being extended (Dillon and Dorman, 2010). Many of these proteins are abundant basic proteins similar to histones and were found to influence chromatin structure and gene transcription. Accordingly, they were earlier named “histone like” proteins (Drlica and Rouviere-Yaniv, 1987; Dorman and Deighan, 2003). This group includes the highly conserved HU (heat unstable), the H-NS (histone-like nucleoid-structuring), IHF (integration host factor) and FIS (factor for inversion stimulation) (Dillon and Dorman, 2010). By the use of a bioinformatics approach it has been estimated that the bacterial nucleoid contains approximately one NAP per 100 bp (Li et al., 2009). According to their architectural mode of action toward DNA, three classes of architectural proteins are distinguished: wrappers, benders, or bridgers (Luijsterburg et al., 2008).

Importantly, there is no sequence or structural similarity between the prokaryotic histone-like proteins and eukaryotic histones (Macvanin and Adhya, 2012). The histone-like HU, H-NS, IHF and FIS proteins bind to AT-rich regions and shape the local structure of DNA upon binding (Browning et al., 2010). In contrast to histones that bind to both coding and non-coding DNA, the binding of these proteins occurs mostly in non-coding regulatory regions of the genome as shown by *in vivo* protein occupancy display (Grainger et al., 2006; Vora et al., 2009).

By electron microscopy, isolated nucleoids of *Escherichia coli* (*E. coli*) were shown to be organized as rosettes with a compact central core from which supercoiled DNA loops with an average size of 10 kbp were observed to radiate (Delius, 1974; Postow et al., 2004). The loops comprise topologically isolated domains with boundaries set by different NAPs such as H-NS and FIS, that can cross-link either different genomic loci or one locus with a membrane (Postow et al., 2004; Travers and Muskhelishvili, 2005; Luijsterburg et al., 2008). At a higher organizational level, the *E. coli* genome is folded into a structure containing four so-called macro-domains with specific NAPs and two less structured regions (Espeli et al., 2008). In *Caulobacter crescentus*, some domain specific NAPs are involved in control of replication and distribution of nucleoids (Dame et al., 2011), while others were shown to regulate the position of chromosomes and the initiation of cytokinesis (Mohl et al., 2001).

NAPs have both structural and regulatory roles. They shape the overall organization of nucleoids depending on the external conditions and growth phase (Rimsky and Travers, 2011). The composition of the NAPs is known to change during the cell cycle, in response to growth phase and external conditions such as nutrient supply and stress factors. For example, FIS is a bending NAP with high levels in growing cells, but it is absent under conditions of slow growth and in cells of the stationary phase (Dillon and Dorman, 2010). In contrast, Dps (DNA protection from starvation), whose expression is regulated by FIS and other NAPs, accumulates at the end of the stationary phase mediating the formation of stable and highly ordered nucleoprotein complexes, also termed biocrystals, that are important for the protection of DNA during stress (Wolf et al., 1999).

In addition to their dynamic functions as structural proteins most NAPs serve dual or multiple purposes and also have specific functions (Dillon and Dorman, 2010; Dame et al., 2011). The HU protein was shown to form transcription foci that are spatially confined aggregations of RNA polymerases (Berger et al., 2010). Other NAPs such as CRP (cyclic AMP regulatory protein) act as transcription factors of specific genes (Nasser et al., 2001; Rimsky and Travers, 2011). The NAP repertoire has considerable impact on global gene expression and in many cases NAPs regulate gene expression by mutually antagonistic activities (Dillon and Dorman, 2010).

Taken together, in contrast to the eukaryotic chromatin, the composition of bacterial nucleoids is more diverse and dynamic. The composition of the NAP fraction is regulated mainly at the level of NAP gene expression whereby NAPs can regulate both the transcription of genes encoding other NAPs and/or their own genes (Travers and Muskhelishvili, 2005).

## DNA organization in plastids

### The plastid genome—size, copy number, and topology

The size of the plastid genome of photosynthetically active algae and higher plants ranges from 120 to 190 kbp depending on the species (Wicke et al., 2011), e.g., in *Arabidopsis thaliana* it is 154 kbp (Sato et al., 1999). The percentage of coding sequence ranges from 50% in the green alga *Chlamydomonas reinhardtii* (Maul et al., 2002) to 93.5% in the red alga *Cyanidioschyzon merolae* (Misumi et al., 2005). Each plastid contains multiple copies of the genome which are distributed among a variable number of nucleoids. Despite the growing number of proteins shown to play roles in DNA replication and maintenance (Maréchal and Brisson, 2010), the mechanism of ptDNA replication is yet not well understood and might depend on the developmental stage of plastids (Nielsen et al., 2010). In fact, several mechanisms of DNA replication were proposed and one involves a chloroplast-targeted RecA protein (Rowan et al., 2010). Of particular importance for ptDNA levels is the activity of an organelle targeted DNA polymerase sharing homology with bacterial DNA polymerase I (Moriyama et al., 2011). In some maize mutants with mutations in the gene encoding the organelle targeted DNA polymerase ptDNA accumulation was observed to be approximately 100-fold reduced (Udy et al., 2012).

The number and positions of nucleoids were shown to depend on the developmental stage of the plastids (Boffey et al., 1979; Kuroiwa et al., 1981). In a recent study on *Beta vulgaris* 12–330 plastid chromosomes per organelle with about 4–7 copies per nucleoid were determined (Rauwolf et al., 2010). It had been suggested long ago that nucleoids even within one plastid contain varying amounts of DNA (Kowallik and Herrmann, 1972). The number of genome copies per plastid changes during chloroplast development (Boffey et al., 1979; Baumgartner et al., 1989), in Arabidopsis ranging from more than 100 in rapidly dividing cells to 20 or fewer in mature cells (Zoschke et al., 2007). Detailled information on plastid DNA copies per cell and per plastid in different plants and in different tissues and stages of development are presented in a recent review (Liere and Börner, 2013). There is controversial information on the DNA content of mature and senescing chloroplasts. Oldenburg and Bendich (2004) reported that mature chloroplasts do not contain DNA, being in contradiction with many other reports (Liere and Börner, 2013). In a recent article a reappraisal of this issue is presented using a combination of high resolution fluorescence microscopy, transmission electron microscopy and real-time quantitative PCR. Thereby the authors demonstrated that considerable levels of DNA and nucleoids are even detectable in plastids of ageing and senescent leaves in different species (Golczyk et al., 2014). The discrepancies between these studies and the former studies of Bendich and co-workers (Rowan et al., 2004) were proposed to be due to methodological insufficiencies of the experimental approaches. Indeed, it is rather unlikely that chloroplasts before entering the degradative phase of late senescence lack DNA, because the D1 protein of the photosynthetic apparatus is known to have a high turnover requiring a continuous re-synthesis (Melis, 1999). The high demand for new synthesis cannot be met by an extremely high stability of plastid mRNAs as claimed by Oldenburg et al. (2014) in their response to the article of Golczyk et al. (2014). In fact, plastid genes are actively transcribed in senescing barley leaves as shown by run-on assays (Krause et al., 1998; Krupinska and Humbeck, 2004). When dark-induced senescence is reverted by light, in particular the transcriptional activities of photosynthesis associated plastid genes were shown to increase again (Krause et al., 1998).

The plastid genome can be divided into four major regions: (1) The large single copy region (LSC) which in Arabidopsis comprises as much as 54% of the genome, (2) the small single copy region (SSC) making up 12% of the plastid genome in Arabidopsis, and (3) the two inverted repeats, IR_A_ and IR_B_, which contain the same genetic information in inverse orientation. Hence the genes contained in these repeats have two copies in the genome. In most plant species the repeats contain three or four ribosomal RNA genes and a number of other genes (Green, 2011). This domain based organization resembles the macrodomain organization of the bacterial genome. However, it is unknown whether, as in the case of bacteria, the different regions of the plastid genome comprise topological and functional units that are associated with specific NAPs as reported for the domains of the bacterial genome.

In contrast to the genomes of the eukaryotic nucleus and of bacteria, the organelle genomes are considered to be highly variable in structure (Bendich, 2004; Oldenburg and Bendich, 2004). Studies employing *in situ* hybridization showed that besides circular chromosomes, linear forms occur in plastids that were proposed to be the major forms in chloroplasts where many small nucleoids are attached to thylakoids (Bendich, 2004). Moreover, the majority of plastid DNA molecules are arranged in multimeric (concatemeric) structures (Deng et al., 1989; Oldenburg and Bendich, 2004; Maréchal and Brisson, 2010). So far, the mechanisms of concatemer formation, linkage and breakage of DNA in plastids are largely unknown (Wicke et al., 2011).

As in bacteria, the DNA in plastids is supercoiled, and plastid DNA topoisomerases play important roles in replication, repair and recombination of DNA (Day and Madesis, 2007). Changes in the DNA topology which especially happen during chloroplast development were proposed to have also dramatic consequences for gene expression (Lam and Chua, 1987; Zaitlin et al., 1989; Salvador et al., 1998).

### Nucleoid associated proteins in plastids

Although several experiments have confirmed that the compact organization of plastid nucleoids is retained by electrostatic interactions between ptDNA and proteins, only a few structural proteins interacting with the ptDNA have been identified so far (Sakai et al., 2004; see Krupinska et al., 2013 for a detailed description of ptNAPs). Most of them have high isoelectric points in accordance with their DNA binding properties (Table 1). Homologs of bacterial HU proteins, namely HU-like proteins, which are known as basic non-specific DNA binding proteins, have been found instead of histones in the nucleus of most dinoflagellates (Sala-Rovira et al., 1991; Wong et al., 2003) and some algae (Bendich and Drlica, 2000). HU-like proteins (HLP) were found to be encoded by the chloroplast genomes of the primitive red alga *Cyanidioschyzon merolae* (Kobayashi et al., 2002) and the green algae *Chlamydomonas reinhardii* (Karcher et al., 2009). These and nuclear encoded HU-like plastid proteins of algae were shown to be functional equivalents of the HU protein by complementation of bacterial mutants lacking HU (Kobayashi et al., 2002). However, in land plants, genes for HU-like proteins have neither been found in any of the sequenced plastid genomes nor in any of the sequenced nuclear genomes (Sato, 2001; Yagi and Shiina, 2014). Novel DNA binding proteins residing in plastids could have evolved from eukaryotic proteins involved in DNA transaction processes in the nucleus (Kodama, 2007; Kodama and Sano, 2007). An intensively studied ptNAP is the plastid envelope DNA binding protein (PEND) having a basic region and a leucine zipper (bZIP) domain. PEND was originally discovered in developing pea chloroplasts (Sato et al., 1993) and shown to tether nucleoids to the inner envelope membrane where replication takes place (Sato et al., 1993, 1998). Interestingly, a PEND:GFP fusion protein was shown to be targeted to the nucleus when the plastid targeting sequence was deleted (Terasawa and Sato, 2009).

Several ptNAPs are multifunctional (Krupinska et al., 2013). One of the most abundant proteins in nucleoids is DCP68 (Cannon et al., 1999) which is identical with sulfite reductase (SiR), an enzyme catalyzing the reduction of sulfite to sulfide (Sato, 2001). SiR was found to bind and compact ptDNA, thereby having a negative effect on *in vitro* replication (Cannon et al., 1999) and transcription (Sekine et al., 2002, 2007; Sato et al., 2003) as well as on chloroplast development (Kang et al., 2010). However, its compacting effect on ptDNA differs from the mode of action of HU-like proteins. In contrast to the DNA packed by HU, DNA tightly packed by SiR, is in an inactive state and is not available for DNA transacting enzymes. SiR was suggested to repress transcriptional activity in non-photosynthetic plastids of spores and seeds (Sato et al., 2003). In some aspects SiR might rather play a similar role as Dps, the bacterial DNA binding protein abundant in starved cells (Dillon and Dorman, 2010). As mentioned above, SiR has the ability to tightly compact DNA, but the impact of this condensation on DNA protection has not been studied so far. On the other hand, considering the association of SiR with nucleoids in mature chloroplasts, SiR may be important beyond the seed stage, putatively playing a role in selective silencing of chloroplast encoded genes.

Novel candidates for architectural ptNAP proteins were identified in a recent study by Melonek and coworkers (2012). A group of six organelle targeted, low molecular weight proteins have a SWIB (switch/sucrose nonfermentable complex B) domain that is typically found in ATP-dependent chromatin remodelers of the nucleus. One of them, SWIB-4, has a histone H1-motif next to the SWIB domain and was shown to bind to DNA. The recombinant SWIB-4 protein was shown to induce compaction and condensation of nucleoids and to functionally complement a mutant of *E. coli* lacking the histone-like nucleoid structuring protein H-NS (Melonek et al., 2012). Interestingly, SWIB domain proteins are also found in *Chlamydophila felis*. This species has a histone 1 like protein (Hc1) and a stand-alone SWIB domain protein, the only type of SWIB proteins found in bacteria. Chlamydiae are a group of bacteria living as endosymbionts and parasites in other bacteria or in eukaryotic cells. Phylogenetic analyses suggested that an ancestral member of the group of Chlamydiae facilitated the establishment of the primary endosymbiosis between cyanobacteria and an early eukaryote (Huang and Gogarten, 2007), and that Chlamydiae have contributed at least 55 genes to plant genomes. Genes encoding members of this subgroup of the SWIB domain proteins (Melonek et al., 2012) are found in the sequenced genomes of all land plants, but not in those of algae. The homology is very high among the sequences found in angiosperms, gymnosperms, mosses and clubmosses (Lycopodiacea).

Other highly abundant proteins of nucleoids are WHIRLY1 (pTAC1) and WHIRLY3 (pTAC11) that have been found in the proteome of transcriptionally active chromosomes (TAC) isolated from Arabidopsis chloroplasts (Pfalz et al., 2006), and that belong to a small family of single-stranded DNA binding proteins specifically found in higher plants. While in most plants one WHIRLY protein is targeted to chloroplasts and one to mitochondria, in Arabidopsis two are targeted to chloroplasts (WHIRLY1, WHIRLY3) (Krause et al., 2005). In other plants such as barley and maize, plastids contain only one WHIRLY protein which is associated to nucleoids (Prikryl et al., 2008; Melonek et al., 2010; Majeran et al., 2012). It has been suggested that WHIRLY1 of barley chloroplasts is located at the periphery of nucleoids, because it is lost during purification of TAC (Melonek et al., 2010). In chloroplasts of transgenic barley plants with an RNAi mediated knockdown of the *WHIRLY1* gene, only few tiny nucleoids are found besides unpacked DNA covering large areas in the organelle (Krupinska et al., 2014). This indicates that WHIRLY1 plays an important role in condensation of plastid DNA of a subset of nucleoids.

**Figure 2:**
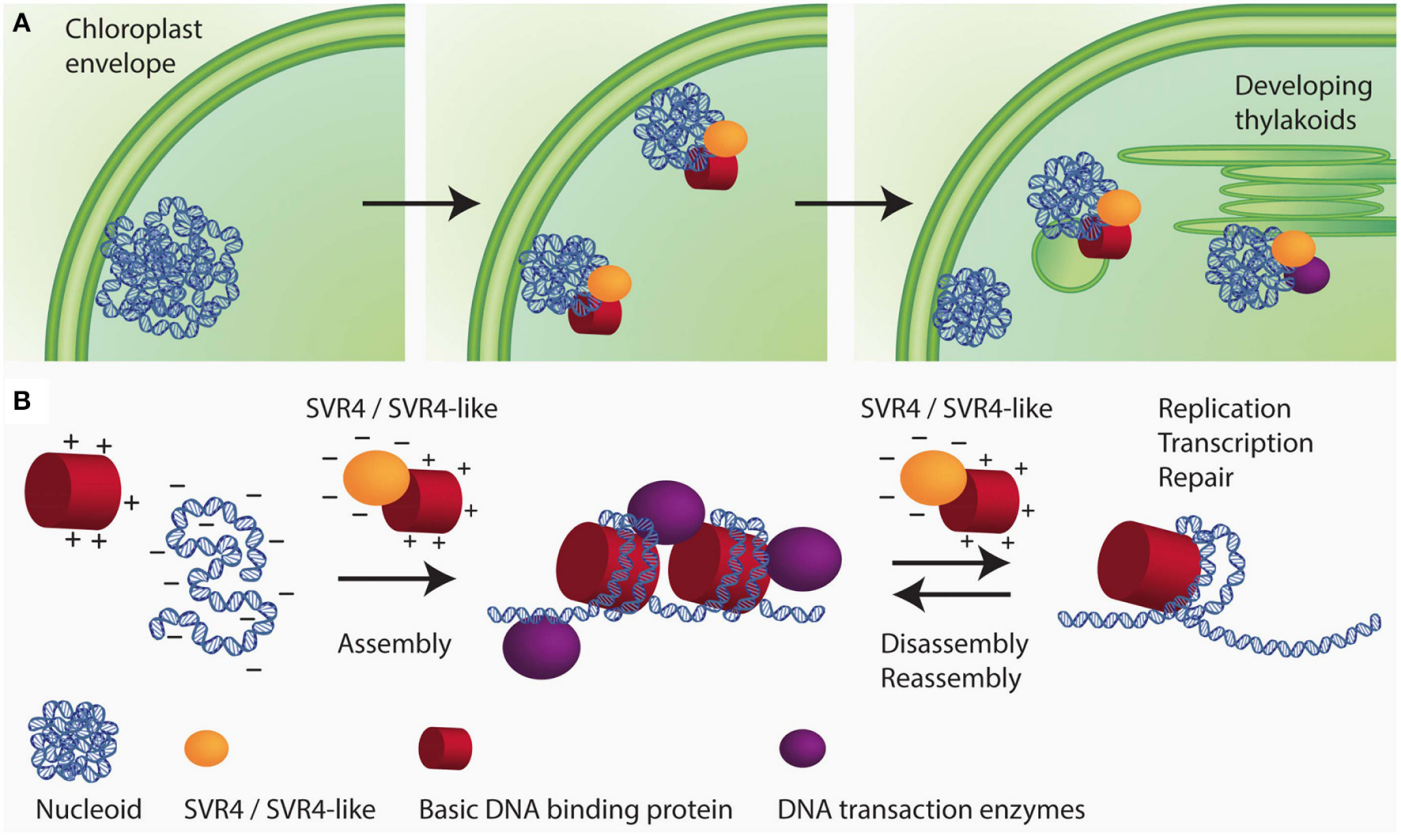
**Schematic drawing of SVR4/SVR4-like functioning as putative chaperones for ptNAPs during chloroplast development. (A)** The spatial arrangement of plastid nucleoids dynamically changes in close relationship with the development of the inner membrane system of the plastids. During chloroplast development the nucleoids decrease in size but increase in number. The segregation process is thought to take place on the envelope membrane and eventually distribute to the thylakoids. **(B)** During the assembly of DNA-protein complexes in the developing chloroplast, there is a high risk of random aggregation due to the fact that very strong interaction occurs between oppositely charged molecular species, i.e., negatively charged DNA and positively charged structural proteins. The negatively charged proteins SVR4 and SVR4-like, transiently interact with positively charged DNA binding proteins, supporting essential DNA transaction processes in chloroplasts.

Additional nucleoid associated proteins specifically found in higher plants are the SVR4 (suppressor of variegation) and SVR4-like proteins, which were originally identified as important proteins for chloroplast development in Arabidopsis (Yu et al., 2011) and were named MRL7 and MRL7-like in another study (Qiao et al., 2011). In the lower land plants, *Physcomitrella patens* and *Selaginella moellendorffii*, only one protein with sequence similarities to both Arabidopsis proteins, SVR4 (MRL7) and SVR4-like (MRL7-like), was found (Qiao et al., 2011). In Arabidopsis, the knockout mutants of either SVR4 or SVR4-like are seedling lethal and can only be grown on media supplemented with sucrose giving rise to pigment deficient plants that are, however, unable to complete their life cycle. SVR4 and SVR4-like are already present in plastids at early stages of chloroplast development. In the absence of either SVR4 or SVR4-like, the nucleoid organization was found to be disturbed. Fewer and larger nucleoids with the tendency to form ring-like structures were detected in the mutants (Powikrowska et al., 2014). In the primary amino acid sequence SVR4 and SVR4-like contain 20% negatively charged glutamic or aspartic acid residues which is a characteristic feature for chaperone proteins, that might assist in assembly and maintenance of DNA/RNA-protein complexes (Powikrowska et al., 2014). During the assembly and dynamic functioning of DNA/RNA-protein complexes there is a high risk of random aggregation due to the fact that very strong interactions occur between the negatively charged nucleic acids and basic proteins such as histones and ribosomal subunits (Jäkel et al., 2002; Frehlick et al., 2007; Lindström, 2011). Negatively charged proteins have been reported to act as chaperones for exposed basic domains most probably by mimicking the interaction with nucleic acids (Jäkel et al., 2002; Koch et al., 2012). It has been proposed that SVR4 and SVR4-like are putative functional homologs of negatively charged molecular chaperones involved in establishing proper ptDNA-protein interaction in developing chloroplasts (Figure 2). The expression of the genes encoding SVR4 and SVR4-like was reported to be high in growing tissues, i.e., young leaves, flowers and stems (Qiao et al., 2011). Interestingly, the level of the SRV4-like is high in the meristematic tissue at the base of a barley leaf, whereas the level of SRV4 increases with chloroplast development (Powikrowska et al., 2014) indicating that the two proteins might have similar functions, but at different stages of chloroplast development.

In conclusion, it seems that most ptNAPs identified so far are unique to land plants (Table 1). Surprisingly, the maize homologs of SiR and PEND were not detected in the extensive nucleoid proteome of maize plastids, although they were found in unfractionated maize plastids (Majeran et al., 2012). It remains to be determined whether the altered distribution of the proteins reflects differences between the different groups of plants or whether it is caused by the method used for preparation of nucleoids. Pfalz and Pfannschmidt (2013) reported that also most of the nucleoid proteins found to be associated with PEP do not have orthologous proteins in the green alga Chlamydomonas indicating that also the prokaryotic transcription machinery has been altered during evolution of land plants. A striking feature of some plastid DNA binding proteins, such as SiR and also CND41, is their multifunctionality (Murakami et al., 2000; Krupinska et al., 2013). A unique example for a multifunctional ptNAP is WHIRLY1. Besides its impact on compactness of a subset of chloroplast nucleoids (Krupinska et al., 2014), WHIRLY1 (pTAC1) has been reported to affect RNA splicing in plastids (Prikryl et al., 2008; Melonek et al., 2010), to be important for DNA stability (Maréchal and Brisson, 2010) and to act furthermore as a transcription factor in the nucleus (Desveaux et al., 2000; Grabowski et al., 2008; Xiong et al., 2009; Krupinska et al., 2014). It remains to be investigated whether the architectural role of WHIRLY1 is connected to its other functions.

## Dynamics of nucleoid organization during chloroplast development

**Figure 3:**
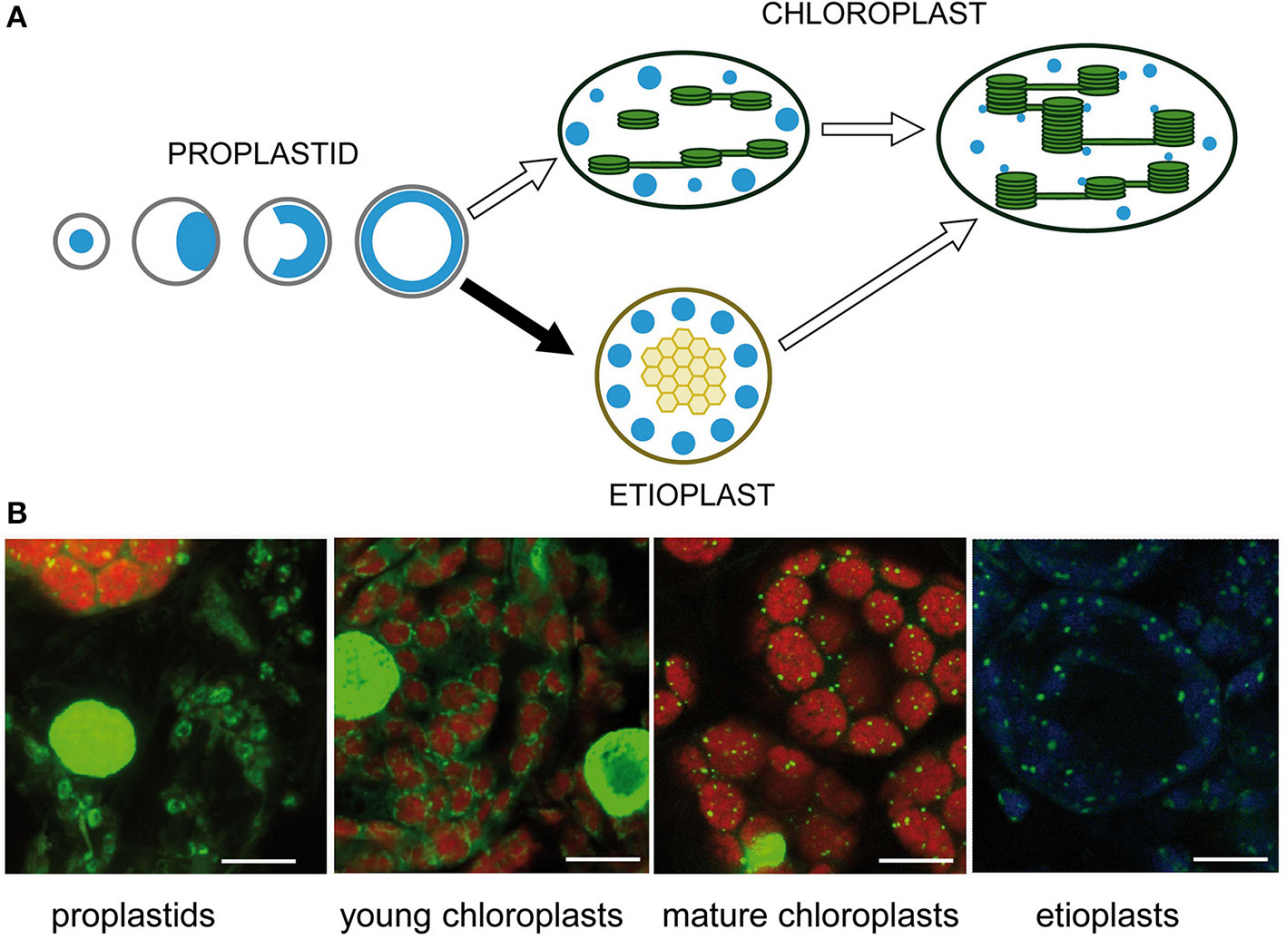
**Nucleoid organization during chloroplast development. (A)** Schematic drawing adapted from Sakai et al. (2004). **(B)** Detection of nucleoids by fluorescence microscopy of barley leaf sections stained with SYBR Green. The left image shows undifferentiated rudimentary plastids in white parts of a striped leaf of the mutant *albostrians* besides a green part containing chloroplasts. Chloroplasts were analyzed in sections of primary foliage leaves of barley seedlings grown for either 5 or 7 days in the light. Etioplasts were analyzed in primary foliage leaves of seedlings after 5 days in darkness. The bar represents 10 μm.

The number and positions of nucleoids were shown to depend on the developmental stage of the plastids (Kuroiwa et al., 1981; Miyamura et al., 1986). Intensive remodeling of nucleoids occurs during the development of proplastids to photosynthetic competent chloroplasts and during interconversions between different plastid types (Hashimoto, 1985; Kuroiwa, 1991; Chi-Ham et al., 2002). Proplastids contain a cluster of nucleoids located in the center of the plastids. At the beginning of seed germination, these nucleoids are considered to move to the envelope, where extensive DNA amplification takes place, and eventually the enlarged nucleoids form a spherical ring (Figure 3A). Upon illumination, during transition from proplastids to chloroplasts, small nucleoids are distributed along developing thylakoid membranes (for review see Sakai et al., 2004). Sections from barley primary foliage leaves were stained with SYBR Green to show typical stages of nucleoid organization. At the border between white and green stripes of a heterozygous leaf of the mutant *albostrians* small undifferentiated and photosynthetically inactive plastids were found besides chloroplasts. As observed in proplastids of leaf primordial of imbibited wheat seeds (Miyamura et al., 1986), the nucleoid in the plastids of white *albostrians* leaves and leaf stripes appears to be ring-shaped. The ring-shaped nucleoid is typical for proplastids developing in darkness. In a basal segment from a primary foliage leaf of seedlings grown for 5 days in the light, developing chloroplasts were found to be organized as a necklace of pearls in the peripheries of the organelles indicating a light-dependent disintegration of the nucleoid ring as proposed previously (Miyamura et al., 1986). In the upper part of a leaf from 7 days old seedlings, mature chloroplasts with many tiny nucleoids attached to thylakoids were found. During development of barley seedlings in darkness, proplastids differentiate into etioplasts, where a few large nucleoids are found that might be distributed at the periphery of prolamellar bodies (Figure 3B). Temporal changes of nucleoid structure have also been studied intensively in variegated leaves of Arabidopsis mutants, e.g., *var2* (Sakamoto et al., 2009). Plastids in white leaf sectors were observed to contain few large nucleoids. During chloroplast development nucleoids were observed to become smaller in size, more dense and more abundant (Sakamoto et al., 2009). The only protein so far identified to be involved in the distribution of nucleoids, YlmG1 (Table 1), is of prokaryotic origin. Overexpression or knockdown of the gene was shown to impair nucleoid partitioning (Kabeya et al., 2010).

Attachment of nucleoids to membranes was proposed to be important for organization, replication and transcription of ptDNA (Sato et al., 1993; Sato, 2001). In chloroplasts, formation of thylakoids seems to be tightly linked with nucleoid morphology and distribution (Kobayashi et al., 2012). Nucleoid structure and transcriptional activity are not affected in mutants developing residual thylakoids with altered lipid composition and impaired photosynthetic machinery (Kobayashi et al., 2012). These studies suggest that the formation of the thylakoid system and the attachment of nucleoids to these membranes precede the assembly of the photosynthetic machinery. On the other hand, ptDNA displays reduced compaction in plastids of the yellow leaf tissue of mutants with silencing of the *CHLD* (Mg chelatase subunit D) and *CHLI* (Mg chelatase subunit I) genes. These mutants possess thylakoids but lack grana stacks and are devoid of the photosynthetic complexes resulting in compromised photosynthesis (Luo et al., 2013). Taken together, these studies demonstrate that thylakoid formation during chloroplast development and alterations in shape and distribution of nucleoids are interconnected processes.

## Functional implications of the thylakoid association of nucleoids in chloroplasts

Interestingly, architectural reorganization of nucleoids during light-dependent chloroplast differentiation is correlated with a switch in polymerase usage: transcription of PEP (plastid encoded polymerase) -dependent genes increases, whereas at the same time the expression of NEP (nuclear encoded polymerase) -dependent genes decreases (Liere et al., 2011). It was recently proposed that the structural establishment of the transcriptional subdomain within the nucleoid represents a bottleneck in chloroplast development (Pfalz and Pfannschmidt, 2013). In this context it has been proposed that the assistance of DNA/RNA-protein assembly factors SVR4 and SVR4-like is required for expression of a set of chloroplast encoded genes involved in chloroplast formation (Qiao et al., 2011; Powikrowska et al., 2014).

Of particular importance for the activity of nucleoids is their association with the thylakoid membranes where the photosynthetic machinery undergoes changes in composition in response to environmental conditions. A prerequisite for remodeling of the photosynthetic apparatus is the regulation of plastid gene transcription in response to light-dependent changes in the redox state of the photosynthetic apparatus (Pfannschmidt et al., 1999). Thereby the composition of the photosynthetic apparatus can continuously be adjusted to the ever changing environmental conditions. Recent research in Chlamydomonas has clearly shown that chloroplast nucleoids are able to sense the redox state and that also the DNA replication activity can be adjusted accordingly (Kabeya and Miyagishima, 2013). SVR4 seems to be among the nucleoid proteins that are able to sense the redox state and to modulate the nucleoid architecture in response to redox changes. SVR4 was reported to possess disulfide reductase activity *in vitro* and to interact *in vivo* with thioredoxin Z (TrxZ), as well as with the two nucleoid associated superoxide dismutases FSD2 and FSD3 (Qiao et al., 2013; Yua et al., 2013). TrxZ regulates the redox state of proteins in response to light and has been shown to be associated with PEP (Steiner et al., 2011; Pfalz and Pfannschmidt, 2013) and to be required for transcriptional activity (Arsova et al., 2010; Schröter et al., 2010). FSD2 and FSD3 are two iron superoxide dismutases found in the chloroplast nucleoid associated with PEP. Both proteins were shown to act as ROS scavengers within the nucleoids (Myouga et al., 2008). It is likely that the redox state of nucleoid associated proteins is regulated by electrons provided from the photosynthetic machinery. It is interesting to note that the enzymatic activity of SiR was shown to be regulated by photoreduced ferredoxin. The DNA binding of SiR did not affect the enzymatic activity suggesting that both ferredoxin and sulfites are accessible to SiR within the nucleoids (Sekine et al., 2007). It remains, however, unknown whether the redox activity of SiR has an impact on DNA binding.

In this context, proteins found to be located at the interface between nucleoids and the thylakoid membrane are of particular interest. MFP1 was proposed to anchor nucleoids to thylakoids in chloroplasts (Jeong et al., 2003). WHIRLY1/pTAC1 is another nucleoid associated protein (Pfalz et al., 2006) associated with thylakoid membranes. Prikryl et al. (2008) showed that the attachment to thylakoid membranes is disrupted by DNaseI. WHIRLY1 was shown to form 24-mer complexes (Cappadocia et al., 2010, 2012) and was proposed to function analogously as the oligomeric NONEXPRESSOR OF PR1 (NPR1) in the cytoplasm (Foyer et al., 2014). Upon changes in the redox state of the photosynthetic machinery the complexes might get monomerized and the monomer might change gene expression in the nucleus. In accordance with this model the WHIRLY3 protein in Arabidopsis chloroplasts was identified among the redox-sensitive proteins (Ströher and Dietz, 2008). Another protein found to be distributed between thylakoids and the nucleoid is pTAC16. Its phosphorylation in response to redox-changes of the photosynthetic apparatus was suggested to regulate membrane-anchoring functions of the nucleoid (Ingelsson and Vener, 2012). It seems that the nucleoid containing besides WHIRLY1 further central proteins shown to be involved in plastid-to-nucleus signaling such as GUN1 (Koussevitzky et al., 2007) and PRIN2 (Kindgren et al., 2012; Barajas-López Jde et al., 2013), is the place where redox signals known to induce changes in nuclear gene expression are integrated.

Transgenic plants with different levels of nucleoid/thylakoid associated proteins might help to elucidate the roles of these proteins in linking the activity of the photosynthetic machinery to organization and expression of plastid genes as well as the expression of nuclear genes.

## Mechanisms underlying the restructuring of plastid nucleoids

In the nucleus the availability of DNA for transcription is regulated mainly by posttranslational modifications, whereas in bacteria regulation of transcription involves the exchange of DNA binding proteins (Luijsterburg et al., 2008) and changes in the compaction of the nucleoid (Berger et al., 2010). It seems, that in plastids the architecture of the nucleoids is regulated by both kinds of mechanisms. Both in the nucleus and in plastid nucleoids, posttranslational modifications are important for nucleoid associated processes. For MFP, SiR and SWIB-4 it was shown that the binding to DNA is regulated by phosphorylation. The three proteins seem not to bind to DNA when they are phosphorylated (Chi-Ham et al., 2002; Jeong et al., 2004; Melonek et al., 2012). Several kinases were found to be associated with nucleoids, e.g., the fructo-kinase like protein FLN1/2, the casein kinase CK-II, as well as two atypical ABCK1 type kinases (Lundquist et al., 2012). Probably, other posttranslational modifications are likely to play important roles as well. Counterparts of enzymes known to be involved in histone modifications in the nucleus, such as the Arabidopsis SET-domain proteins ATXR5 and ATXR6 involved in methylation (Raynaud et al., 2006; Jacob et al., 2009) and de-acetylases were found in plastids (Chung et al., 2009).

It remains to be shown whether the different packaging of DNA in different regions of the nucleoid changes during development and in response to environmental cues. Whether, however, the central body of nucleoids with dense packaging (Sakai et al., 2004) can be compared with eukaryotic heterochromatin (Sato, 2001) remains questionable. It rather seems that DNA packaging is beneficial for a high transcriptional activity as it was described for bacteria as well (Dillon and Dorman, 2010; Krupinska et al., 2013).

## Concluding remarks

The comparison of DNA organization in plastids, nucleus and bacteria shows that the shaping and organization of plastid nucleoids involves novel organelle specific mechanisms resembling those acting on eukaryotic chromatin besides mechanisms described for eubacterial nucleoids. During evolution of plants, the architectural proteins of bacterial nucleoids have been lost and replaced by new proteins. Some of these are enzymes that have acquired an additional function as DNA binding proteins. Others might have been contributed by Chlamydiae which facilitated establishment of the primary endosymbiosis between an early eukaryote and the cyanobacterial ancestor of plastids. These proteins do not exhibit sequence or structural conservation with the eukaryotic histones, but similar to the histones they might be regulated by posttranslational modifications.

In comparison to eukaryotic chromatin, nucleoids of plastids have as those of bacteria a more open structure, that allows easy access for DNA transaction enzymes. The enrichment of enzymes involved in RNA processing and translation in the nucleoid fraction suggests that transcription, RNA processing and translation are tightly connected with each other.

Similarly to bacteria, also in plastids, membranes seem to play a key role in the organization and maintenance of nucleoids. In chloroplasts, the proximity of nucleoids and photosynthetic machinery as well as the presence of several redox active proteins in nucleoids, allows for a tight coordination of photosynthesis and nucleoid function, i.e., replication and gene expression. It is striking that not only particular enzymes involved in gene expression but also architectural proteins are controlled by redox signals. Thereby these proteins might have a tremendous impact on the different enzymatic activities associated with nucleoids; in particular replication, transcription and DNA repair.

The architectural organization of the plastid genetic machinery is not well understood. Since principles underlying the dynamic shaping of genomes are uniform in all forms of life, the knowledge about DNA organization in bacteria and eukaryotes can be used in future studies on the dynamic architecture of chloroplast nucleoids.

### Conflict of interest statement

The authors declare that the research was conducted in the absence of any commercial or financial relationships that could be construed as a potential conflict of interest.
